# Draft Genome Sequences of the *Enterococcus faecium* Strain LCT-EF258

**DOI:** 10.1128/genomeA.00147-12

**Published:** 2013-02-14

**Authors:** De Chang, Yuanfang Zhu, Xiangqun Fang, Tianzhi Li, Junfeng Wang, Yinghua Guo, Longxiang Su, Yan Liu, Xuege Jiang, Li Wang, Na Guo, Changting Liu

**Affiliations:** a Nanlou Respiratory Diseases Department, Chinese PLA General Hospital, Beijing, China; b BGI-Shenzhen, Shenzhen, People’s Republic of China

## Abstract

The space environment has been shown to affect microbes by altering various features, including morphology, growth rate, metabolism, virulence, drug resistance, and gene expression and mutation. Here we present the draft genome sequence of the *Enterococcus faecium* strain LCT-EF258, derived from the *E. faecium* strain CGMCC 1.1736, which was exposed to 17-day space flight.

## GENOME ANNOUNCEMENT

The space environment has been reported to cause biological alterations in microorganism characteristics such as growth, drug resistance, and virulence (1–4). *Enterococcus faecium* was originally defined as a group D streptococcus until DNA analysis in 1984 showed the differences in streptococci. These organisms can be part of the normal flora in human and animal intestines as well as pathogens causing nosocomial infections (1, 5). *E. faecium* is a Gram-positive bacterium belonging to the family *Enterococcaceae* (6). We employed *E. faecium* as a model to investigate the effects of space conditions on microbes. The *E. faecium* strain LCT-EF258 was derived from the strain CGMCC 1.2136, which was loaded onto the ShenZhouVIII spacecraft, which flew in space from 1 November to 17 November 2011. The whole-genome sequence of the strain LCT-EF258, which has been exposed to space flight, was determined using Illumina second-generation sequencing technology (7).

The genomic DNA of *Enterococcus faecium* LCT-EF258 was sequenced by the Illumina Hiseq2000, and shotgun sequence libraries were prepared for two types with 500 bp and 6 kb. By a series of filtering criteria, a total of 550 Mb of sequence reads was generated (read length, 90 bp). With SOAPdenovo software (8), we joined 114 contigs with a total length of 2,717,416 bp and 26 scaffolds (≥500 bp in size) with 90-kb gaps. About 190-fold coverage was achieved.

Open reading frames (ORFs) were identified with Glimmer (9), and 2,824 protein-coding sequences (CDSs) with an average gene length of 861 bp were predicted. The putative encoded proteins were compared with sequences present in public databases by using BLASTP. Other putative coding regions, either genes or pseudogenes, were identified by BLAST. tRNAs were identified by using the program TRNA-SCAN (10). rRNAs and other small RNAs were identified by BLASTN searches of the intergenic regions versus RNA-specifying genes in other published genome sequences.

The gene functional annotations were yielded by BLASTP using all predicted protein sequences, and the graphical display of the annotated genome was obtained with the KEGG (11), Clusters of Orthologous Groups (COG), Swiss-Prot, TrEMBL, Gene Ontology (GO), and NR databases. For the COG database, the genes were clustered mainly into the “carbohydrate transport and metabolism” and “general function prediction” classes. In addition, 1,901 protein families were found by comparing protein sequences with sequences in protein databases using BLASTP.

### Nucleotide sequence accession number.

The whole-genome sequence of *E. faecium* LCT-EF258 has been deposited at DDBJ/EMBL/GenBank under the accession number ANAJ00000000. The versions described in this paper are the first versions.

## References

[1] Juergensmeyer MA Juergensmeyer EA Guikema JA 1999 Long-term exposure to spaceflight conditions affects bacterial response to antibiotics. Microgravity Sci. Technol. 12:41–47 11543359

[2] Leys NM Hendrickx L De Boever P Baatout S Mergeay M 2004 Space flight effects on bacterial physiology. J. Biol. Regul. Homeost. Agents 18:193–199 15471227

[3] Wilson JW Ott CM Höner zu Bentrup K Ramamurthy R Quick L Porwollik S Cheng P McClelland M Tsaprailis G Radabaugh T Hunt A Fernandez D Richter E Shah M Kilcoyne M Joshi L Nelman-Gonzalez M Hing S Parra M Dumars P Norwood K Bober R Devich J Ruggles A Goulart C Rupert M Stodieck L Stafford P Catella L Schurr MJ Buchanan K Morici L McCracken J Allen P Baker-Coleman C Hammond T Vogel J Nelson R Pierson DL Stefanyshyn-Piper HM Nickerson CA 2007 Space flight alters bacterial gene expression and virulence and reveals a role for global regulator Hfq. Proc. Natl. Acad. Sci. U. S. A. 104:16299–16304 17901201 10.1073/pnas.0707155104 PMC2042201

[4] Rosenzweig JA Abogunde O Thomas K Lawal A Nguyen YU Sodipe A Jejelowo O 2010 Spaceflight and modeled microgravity effects on microbial growth and virulence. Appl. Microbiol. Biotechnol. 85:885–891 19847423 10.1007/s00253-009-2237-8 PMC2804794

[5] Murray BE 1990 The life and times of the enterococcus. Clin. Microbiol. Rev. 3:46–65 2404568 10.1128/cmr.3.1.46 PMC358140

[6] Franz CM Stiles ME Schleifer KH Holzapfel WH 2003 Enterococci in foods—a conundrum for food safety. Int. J. Food Microbiol. 88:105–122 14596984 10.1016/s0168-1605(03)00174-0

[7] Shendure J Ji H 2008 Next-generation DNA sequencing. Nat. Biotechnol. 26:1135–1145 18846087 10.1038/nbt1486

[8] Li R Zhu H Ruan J Qian W Fang X Shi Z Li Y Li S Shan G Kristiansen K Li S Yang H Wang J Wang J 2010 De novo assembly of human genomes with massively parallel short read sequencing. Genome Res. 20:265–272 20019144 10.1101/gr.097261.109 PMC2813482

[9] Delcher AL Bratke KA Powers EC Salzberg SL 2007 Identifying bacterial genes and endosymbiont DNA with Glimmer. Bioinformatics 23:673–679 17237039 10.1093/bioinformatics/btm009 PMC2387122

[10] Lowe TM Eddy SR 1997 tRNAscan-SE: a program for improved detection of transfer RNA genes in genomic sequence. Nucleic Acids Res. 25:955–964 9023104 10.1093/nar/25.5.955 PMC146525

[11] Kanehisa M Goto S Furumichi M Tanabe M Hirakawa M 2010 KEGG for representation and analysis of molecular networks involving diseases and drugs. Nucleic Acids Res. 38:D355–D360 19880382 10.1093/nar/gkp896 PMC2808910

